# Can mHealth interventions contribute to increased HPV vaccination uptake? A systematic review

**DOI:** 10.1016/j.pmedr.2020.101289

**Published:** 2020-12-28

**Authors:** Onaedo Ilozumba, Paula Schmidt, Johannes C.F. Ket, Monique Jaspers

**Affiliations:** a Vrije Universiteit Amsterdam, Faculty of Sciences, The Boelelaan 1105, 1081 HV Amsterdam, The Netherlands; b Medical Library, Vrije Universiteit, De Boelelaan 1117, 1081 HV Amsterdam, The Netherlands; c Centre for Human Factors Engineering of Interactive Health Information Technology (HIT-lab), Department of Medical Informatics, J1B-116, Amsterdam Public Health Research Institute – AmsterdamUMC, Location Academic Medical Center, PO Box 22700, Amsterdam, The Netherlands

**Keywords:** Human papillomavirus, mHealth, Vaccine uptake, Adolescent health, Immunization

## Abstract

The objective of this review was to synthesize existing evidence on mobile health (mHealth) interventions geared at improving HPV related knowledge, HPV vaccination intent and HPV vaccination uptake. Between November and December 2019, systematic searches were performed in the databases PubMed, Ebsco/CINAHL, Ebsco/PsycINFO, and Clarivate Analytics/Web of Science Social Science Citation Index (SSCI). We identified 805 articles of which 92 were eligible for inclusion after abstract screening. 19 articles met the inclusion criteria of the review based on full article review and all but one of the included interventions were conducted in the United States. mHealth interventions ranged from text messages, phone calls to interactive voice recordings and software on tablets. All four interventions which attempted to improve HPV knowledge and vaccination intent, reported positive findings, however, only two interventions reported statistically significant improvements between intervention and control groups. 14 interventions reported an increase in HPV vaccination uptake and vaccination series completion among the intervention groups; including when mHealth interventions were compared to control groups or non-mHealth interventions. Some factors noted in the successful interventions included frequency of messages, combinations of multiple interventions including in-person education session, written educational materials, texts alongside call or emails; tailored messages as well as participant’s intent to vaccinate at baseline. mHealth interventions potentially show promise as tools in improving short-term vaccination knowledge, intent, and uptake of HPV vaccination. There is however a need to refine components which can promote mHealth interventions’ success as well as for the evaluation of such interventions in different contexts and over time.

## Highlights

• 19 studies which utilized mHealth interventions to improve HPV vaccination outcomes.
• Interventions recorded positive but not always statistically significant outcomes.
• Limitations of the included studies indicate that further research is needed.

## 1 Introduction

The Human Papillomavirus (HPV) is the most common sexually transmitted infection (STI) and most sexually active adults will contract this STI at some point in their lives (Forman et al., 2012). The virus has direct causal links to cancer of the cervix, vulva, vagina, anus, penis, and oropharynx (Marur et al., 2010, Muñoz, 2000, zur Hausen, 2009, zur Hausen, 1991). This link is particularly well researched in the case of cervical cancer, where approximately 90% of cervical cancers are attributed to HPV infections (de Martel et al., 2017). In 2006, the first effective HPV vaccine became available and has since been recommended as a routine vaccination for children and young adults before the onset of sexual activity (Barnard et al., 2019, Ehrhardt, 2007, WHO, 2013). By 2018, 81 countries had revised their immunization policies and integrated HPV vaccines into the national immunization program (WHO, 2018). Despite this policy change, the risks associated with the virus and benefits conferred by the vaccination, HPV vaccination uptake remains suboptimal in most countries (Bruni et al., 2016). Rates of HPV vaccine uptake vary among low and middle-income countries, but there have been reported increases in vaccination rates since 2014 when it was estimated that only 1% of the population was vaccinated (LaMontagne et al., 2017). While uptake is higher in high-income countries, which first implemented HPV vaccinations, reported rates still vary greatly between countries (Gallagher et al., 2018).

Research on health-seeking behavior and general vaccination uptake has shown that lower levels of health-seeking and vaccination uptake are associated with demographic characteristics (socioeconomic status, ethnicity, religion), as well as barriers such as costs, perceived susceptibility, multiple required visits and subjective norms. (Barnard et al., 2019, Dodd et al., 2016, Forster et al., 2010, Holman et al., 2014, Newman et al., 2018, Schurink and de Melker, 2017, Walling et al., 2016). Low vaccination rates are also closely linked to a lack of awareness and knowledge of the risks associated with HPV (Loke et al., 2017). Multiple health promotion interventions are utilized to address the problem of low vaccine uptake, including digital technologies.

Digital technologies have been described as essential tools to improve health outcomes (WHO, 2019), as they enable information access at all times, provide support and guidance, facilitate quick and easy communication, increase independence and self-care and ultimately promote health (Kolff et al., 2018). One often utilized aspect of digital technologies is mobile health or mHealth, which is often defined as the delivery of health information on a mobile phone or handheld device (Bashshur et al., 2011). One comprehensive definition describes mHealth as emerging mobile communications and network technologies for healthcare, which involves wireless communications (Istepanian and Swamy Laxminarayan, 2006, p.30). mHealth can utilize the basic voice and SMS functions of wireless devices as well as more complex functions and applications (WHO, 2011). Worldwide, an estimated 5 billion people are mobile phone users (Oliver-Williams et al., 2017a). Researchers have highlighted the potential of utilizing these technologies as interventions to increase vaccination uptake rates (Atkinson et al., 2019, Jacobson Vann et al., 2018).

There have been efforts to synthesize interventions geared at addressing HPV vaccination uptake. The most recent by Barnard et.al focused on a range of interventions to increase HPV vaccination rates among college students (Barnard et al., 2019), including websites and paper pamphlets. Although this review did include some studies which utilized mHealth interventions the focus was on their utilization among college students, not mHealth functionalities. To our knowledge, there has not been a review which focused on the use of mHealth in HPV vaccine uptake. This creates a gap in the understanding of what types of mHealth interventions are utilized to improve HPV related knowledge, intent to vaccinate and vaccination uptake or how these programs effectiveness at achieving their goals. As mHealth interventions are likely to continually be used; it is important to review existing evidence. The purpose of this systematic review is to synthesize existing evidence on mHealth interventions geared at improving HPV related knowledge, vaccination intent and vaccination uptake. We aim to understand what mHealth interventions are utilized to improve HPV vaccine uptake, which populations these interventions target and report the observable outcomes.

## 2 Material and methods

The Preferred Reporting Items for Systematic Reviews and Meta-Analyses (PRISMA) was utilized to guide this systematic review of the literature (Moher et al., 2015). Systematic searches were performed (by JK, OI and PS) in the bibliographic databases PubMed, EBSCO/CINAHL, EBSCO/PsycINFO, and Clarivate Analytics/Web of Science Social Science Citation Index (SSCI). PubMed was searched from inception up to November 5, 2019; CINAHL, PsycINFO and Web of Science SSCI were searched from inception up to November 20, 2019. Search terms associated with the three broad topics (1) human papilloma virus, (2) vaccination, and (3) digital intervention, were used as index terms and free-text words. No limit on language or publication date was used. The full search strategies used are presented in Supplementary File 1*.*

All identified articles were screened based on the following inclusion criteria: i) interventions focused on either the target population for the vaccination (including male and/or female adolescents or young adults) or caregivers responsible for decision-making ii) mHealth interventions focused on HPV vaccination including SMS or text messages, mobile applications and phone calls, applications installed on smartphones or tablets iii) interventions focused on one or more of the following three outcomes (a) knowledge-related outcomes, including knowledge of the HPV virus and HPV vaccinations (b) HPV vaccination intentions (c) HPV vaccination uptake. Studies were excluded: a) if they targeted primarily health care professionals, b) did not include an intervention but were either observations of online communication or social media use or focused on the development of messaging.

Two authors (OI and PS), first independently screened 15% of the titles and abstracts from the literature searches to ensure agreement over the application of the inclusion and exclusion criteria. OI and PS then screened all titles and abstracts for inclusion based on the agreed-on criteria. Screening of titles and abstracts was conducted in Rayyna which allowed the authors to easily compare assessments, identify areas of agreement and disagreement (Ouzzani et al., 2016). Two authors (OI and PS) independently extracted two articles to ensure that the extraction tool captured all necessary information for the review. Information extracted included characteristics of participants (e.g. age, socio-economic status, race), study design, intervention description, study outcome, author reflections. Full-text articles were read by two authors (OI and PS) with a third author (MJ) available to resolve any discrepancies. References of included articles were scanned to identify articles for inclusion and no new articles were identified. Risk of Bias was assessed using two assessment criteria specific for randomized and non-randomized trails. The randomized control trials were evaluated using the revised Cochrane risk-of-bias tool for randomized trials (RoB2) (Sterne et al., 2019). For experimental studies without random allocation the Joanna Briggs Institute (JB1) Critical appraisal tools was used (Tufanaru et al., 2020).

## 3 Results

### 3.1 Included studies

**Fig. 1:**
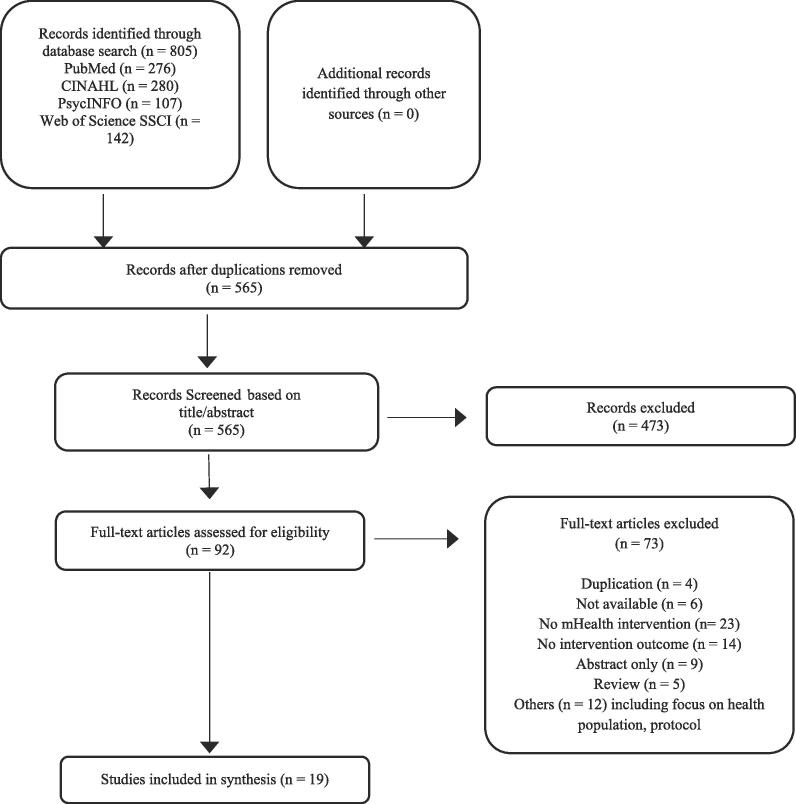
Article selection flowchart.

The search strategy yielded 805 articles of which 565 were duplicates. Due to significant author and title differences, four additional duplications were identified at the stage of full-text selection. Significant author and title differences refer to incidences in which an article with apparently different titles and authors was discovered to be the same manuscript. A total of 92 articles were identified for full-text review and after full-text reading, 73 articles were excluded. Specific reasons for exclusion were duplications (n = 4), manuscript not available (n = 6), no mHealth intervention (n = 23), no intervention outcome as defined by the review (n = 14), literature review (n = 5), abstract only (n = 9). The authors of nine abstracts were contacted, as there was no access of their publication available through the Vrije Universiteit van Amsterdam or the University of Amsterdam. Eight authors provided the requested article, whereas one did not respond. Five articles were not obtained, as neither the authors nor the articles could be found on any database. A flowchart outlining the protocol adopted for this systematic review is displayed in Fig. 1.

All but one of the nineteen studies included were conducted in the USA (Aragones et al., 2015, Bar-Shain et al., 2015, Cassidy et al., 2014, Dempsey et al., 2019, Dixon et al., 2019, Henrikson et al., 2018, Keeshin and Feinberg, 2017, Kempe et al., 2016, Kharbanda et al., 2011bb, Lee et al., 2016, Matheson et al., 2014, Morris et al., 2015, Patel et al., 2014, Rand et al., 2015, Richman et al., 2019, Richman et al., 2016, Szilagyi et al., 2013). One study was conducted in Australia (Tull et al., 2019). Eleven of the included studies were randomized control trials (Dempsey et al., 2019, Dixon et al., 2019, Henrikson et al., 2018, Kempe et al., 2016, Patel et al., 2014, Rand et al., 2015, Rand et al., 2017, Richman et al., 2016, Richman et al., 2019, Szilagyi et al., 2013, Tull et al., 2019). The other nine studies were variations of non-randomized pre-post designs with control groups (Aragones et al., 2015, Bar-Shain et al., 2015, Cassidy et al., 2014, Keeshin and Feinberg, 2017, Kharbanda et al., 2011aa, Lee et al., 2016, Matheson et al., 2014, Morris et al., 2015)

The interventions most frequently targeted parents (Aragones et al., 2015, Bar-Shain et al., 2015, Cassidy et al., 2014, Dempsey et al., 2019, Dixon et al., 2019, Henrikson et al., 2018, Kempe et al., 2016, Kharbanda et al., 2011bb, Morris et al., 2015, Rand et al., 2015, Rand et al., 2017, Richman et al., 2016, Richman et al., 2019). Five interventions focused on adolescents (defined by the articles as 11–22 years old) or young adults (defined by articles as 18–26 years old) (Keeshin and Feinberg, 2017, Kempe et al., 2016, Patel et al., 2014, Richman et al., 2016; Szilagyi et al., 2013). Ten of the interventions targeted both females and males (Dempsey, 2019, Dixon et al., 2019, Henrikson et al., 2018, Keeshin and Feinberg, 2017, Matheson et al., 2014; Morris et al., 2015, Rand et al., 2015, Rand et al., 2017, Tull et al., 2019). Only two studies focused exclusively on females (Kharbanda et al., 2011bb, Szilagyi et al., 2013). Table 1 provides an overview of the included studies.

**Table 1:** Overview of included studies.

Author	Location of Study	Research Design and Bias Assessment	Sample and Sample size	Study Aim	Outcome Measures and Study Results
Aragones et al. (Aragones et al., 2015)	New York, USA	Controlled before and after study Loss-to-follow-up was not addressed in analysis	69 participants: 24 (control) and 45 (intervention). 100% Mexican origin, 80% female and limited English proficiency.	Assessed text message reminders	HPV vaccine series completion (3 doses). Those in the text message group were 15.5 times more likely to complete the vaccination than those in the education-only group (p < 0.001). Vaccination was also associated with parents age and awareness of the vaccine before study participation.
Bar-Shain et al. (Bar-Shain et al., 2015)	Ohio, USA	Single-arm pre-post design Concerns due to lack of a control group	n = 3,933 adolescents (mean age = 14.4) 50% male and 38% black was. 79% had public insurance and 85% overdue on HPV vaccination	Study the impact of direct messages	Completion of HPV, meigicococcal conjugate vaccine (MCV) or tetanus-diphtheria and pertussis (Tdap) vaccination. Receiving one message was associated with a greater likelihood of vaccination compared to receiving two (19.4%) or three messages (p < 0.0001). Receiving a single text (38.8%) and postcards (40.1%) we were more likely to get vaccinated than those receiving a phone call
Cassidy et al. (Cassidy et al., 2014)	USA	Quasi-experimental study High risk due to lack of a control group and missing descriptive variables	n = 23. 96% mothers, 60% white, 83% greater than high school education	Evaluated the impact of educational brochure and telephone reminder strategy	HPV vaccine uptake and completion (3 doses) and satisfaction with the clinical protocol. Parents who received the intervention were 9.4 times more likely to have uptake of the HPV vaccine compared with the historical control group. ﻿Parents who received the intervention were 22.5 times more likely to complete the three-dose series compared with the historical control group. ﻿
Dempsey et al. (Dempsey et al., 2019)	Colorado, USA	Three armed randomized control trial Potential concerns due to bias randomization process, deviations from intended interventions and selection of the reported results	1294 (tailored intervention = 430, untailored = 425, usual care = 439) Mean age 22 years (young adults), 12 years (adolescents); 100% female (young adults), 51% male (adolescents); 85% Hispanic (young adults) and 93% Hispanic (adolescents)	Assessed the effect of a tailored educational digital intervention (CHICOs)	Vaccination intent and receipt of vaccination. There were no differences between study arms in vaccination intention at baseline or post-intervention for either parents or young adults. ﻿
Dixon et al. (Dixon et al., 2019)	Indiana, US	Cluster-randomized trial Potential concerns due to bias randomization process, measurements of outcomes and selection of the reported results	1596: 1059 (control), 537 (intervention). 57% were 11–12 year, 54% African American, 78% on Medicaid, 57%	Tested the effect of a digital HPV vaccine educational intervention	HPV vaccine uptake series initiation (dose 1) and completion (3 doses). HPV vaccination uptake (change in HPV dose status) adolescents at the intervention clinic had nearly double the odds of receiving a dose of the HOPV vaccine (OR:1.82. P < 0.001). Comparing HPV uptake between those who received a tablet and those who did not, adolescents who received the tablet had 3 times greater odds of received ta dose for the HPV vaccine (OR = 3.07; P = 0.003).
Henrikson et al. (Henrikson et al., 2018)	Washington, USA	Randomized control trial Low risk of bias but some concerns related to measurement of outcomes	1805 (1354, intervention group; 451 control group). 46% aged 10 years at randomization, Intervention group: Male 51%, White 65%. Control group: Male 53%, White 63%	Developed, implemented and tested an outreach and reminder intervention	HPV vaccine series completion (3 doses). No significant differences in receipts of the first vaccination. However, the intervention group was more likely to complete the series (10.3%) compared with usual care (6.8%) p = 0.035. ﻿
Keeshin et al. (Keeshin and Feinberg, 2017)	Ohio, USA	Prospective cohort study Concerns due to potential selection bias	255 (28- intervention and 212 control). Intervention group: 22.8 mean age, 82% black, 61% uninsured, 75% male. Control group 223.5 mean age, 81% male, 60% black, 49% uninsured	Evaluated the feasibility and efficacy of text and e-mail reminder–recalls.	﻿Receipt of one and all HPV vaccinations at 6 and 12 months. Patients who were sent a monthly text or e-mail message received ≥ 1 HPV immunization than controls (p < 0.05). More patients completed the 3-dose HPV series in the intervention group than control (p < 0.05).
Kempe et al. (Kempe et al., 2016)	Colorado, USA	Cluster randomized pragmatic trial High risk of bias due to randomization process, deviations from the intervention, missing outcome data and measurement of the outcome	1422 (intervention enrolled = 374, intervention not enrolled = 493, control = 555). Overall sample, mean age was 13. Intervention enrolled 67% male, 48% white and 22% Hispanic. Intervention not enrolled: 65% make, 47% white, 23% Hispanic. Control: 64% make, 53% white and 26% Hispanic	Described parental preference for HPV recall and assessed the effectiveness of preference-based recalls	HPV vaccine uptake series completion (3 doses). Adolescents in the intervention group were more likely to receive vaccines within the recommended dosing intervals for all doses (p > 0.01). The intervention was more effective for younger adolescents (p < 0.01) and reminding the parent and adolescent did not increase effectiveness.
Kharbanda et al. (Kharbanda et al., 2011aa, Kharbanda et al., 2011bb)	New York, USA	Non-randomized experimental study Possible selection bias	1,512 (intervention = 124, control 1 = 308, control 2 = 1,080) Mean age −14. Intervention- 83% Medicaid/SCHIP, 53% English language Control 1–90% Medicaid/SCHIP, 40% English and Control 2 68% Medicaid/SCHIP, 29% English	Implemented and evaluated and text message reminders	Receipt of for the second and third vaccine doses. The intervention group was more likely to receive their next HPV vaccine dose on time–within one month of its due date (p = 0.001).
Lee et al. (Lee et al., 2016)	Minnesota, USA	Non-randomized quasi-experimental High risk due to lack of a control group and limited sampling	30 (all intervention group) 100% female, 43% aged 23–25, 97% unmarried, 63% unemployed, 63% graduated high school.	Tested the feasibility and effect of tailored mHealth intervention	Improved knowledge, attitudes, and beliefs about cervical cancer prevention; increase internet to receive HPV vaccination; increase receipt of the HPV vaccine. Improved knowledge about HPV and HPV vaccination, personal barriers to crucial cancer prevention and screening, cultural-based attitudes toward cervical cancer screening and prevention ad well as self-efficacy toward cervical cancer prevention (p > 0.01).
Matheson et al. (Matheson et al., 2014)	North Carolina, USA	Non-randomized controlled trial Potential selection bias and limited description of study population	312 (intervention group = 37, interested group (enrolled but did not complete opt in) = 43, control group = 232)	Evaluated text message reminders	HPV vaccine series completion rates. The text group performed significantly better on all 4 outcomes than the control group (p < 0.05).
Morris et al. (Morris et al., 2015)	California, USA	Non-randomized controlled trial Some concerns due to missing analysis	5,050 (intervention group = 1,797, enrollment call only = 3,253, nonintervention = 116,356) Intervention group 50% female, enrollment group 48% female and non-intervention – 43% female	Assessed the effectiveness and cost-efficiency of three reminder/ recall methods	HPV vaccination rates. Participants who received a repeated reminder were more likely to be up to date than those in the enrolment phone call only group (24.6% vs 12.4% P < 0.001). Text messages were the most effective reminder method.
Patel et al. (Patel et al., 2014)	North Carolina Utah, Arizona, Washington, Colorado, California, Illinois, USA	Cluster randomized controlled trial Some concerns due to potential bias in the randomization process, deviations from intended intervention and measurement of outcomes	365 (intervention = 180, control = 185). Mean age 23. Intervention – 47% White, 52% completed high school or less and 55% had no health insurance, 77% had 3 or more sexual partners. Control group – 66% White, 49% completed high school or less, 58% had no insurance, 89% had 3 or more sexual partners	Evaluated an automated reminder system	Vaccine series completion. No significant difference in completion rates between intervention and control groups
Rand et al. (Rand et al., 2015)	Rochester, NY	Randomized controlled trial Some concerns due to missing outcome data and selection of reported data	3812 (intervention = 1,893, control = 1,919). 54% male and 59% Medicaid Insurance	Evaluated the effectiveness of text-message based reminder	Receipt of the first dose of HPV vaccine. No statistically significant increase in first dose vaccination for the intervention group
Rand et al. (Rand et al., 2017)	New York, USA	Parallel randomized controlled Low risk of bias.	749 (phone intervention = 178, phone control = 180 text intervention = 191, text control = 200). Phone group (intervention and control) − 65% male, 82% public insurance, 71% Black. Text group (intervention and control) 68 make, 79% public insurance, 61% black	Assessed the effect of phone or text message reminders	HPV vaccine completion rates. In the phone arm, there was no significant difference in rates of HPV doses 1–3 between intervention and control groups. In the text group, participants in the intervention completed the series (49% vs 31% with 3 doses, p < 0.001). Females in the text reminder intervention were more likely that males to complete the series (p < 0.001).
Richman et al. (Richman et al., 2019)	North Carolina, USA	Parallel randomized controlled Low risk of bias	262 (intervention = 129, control = 133) Mean age 20.7. 60% female and 54% white. 86% reported ever having sex	Evaluated the effectiveness of different messaging types	Completion of the second and third dose of HPV vaccine. No significant difference in completion rates between make and females. Mean knowledge score for the intervention group participants was significantly higher at follow up than their baseline score (93% vs 87%, p = 0.01).
Richman et al. (Richman et al., 2016)	North Carolina, USA	Randomized longitudinal study Some concerns due to missing outcome data and selection of the reported results	264 (intervention = 129, control = 128)	Evaluated an educational and reminder strategy	Completion of second and third dose of HPV vaccine. No significant difference in completion dose rates or knowledge for intervention and control group.
Szilagyi et al. (Szilagyi et al., 2013)	New York, USA	Randomized control study High risk of bias due to missing outcome data, measurement of the outcomes and selection of reported data	4115 (Mailed intervention = 1.396, telephone intervention = 1423, control = 1296) Mean age 14 years	Assesse the effect of reminder system	﻿Receipt of vaccinations (Tdap, MCV4 and HPV). For children who were behind on a given vaccine, there was a significant increase in vaccination in both the mailed and telephone interventions (p < 0.05)
Tull et al. (Tull et al., 2019)	Victoria, Australia	Randomized control study Some concerns due to measurement of the outcome and selection of reported results	4386 (Motivational message- 1,442, self-regulations message = 1,418, control = 1.526)	Assessed the effect of SMS reminders	Receipt of HPV vaccination. SMS conditions led to higher vaccination rates at the third school visit than the control condition (p = 0.10). However, there was no significant difference in vaccination rates at the third school visit between the motivation and self-regulatory messages.

Risk of bias assessments conducted for the RCT studies (11 of the 19 studies) showed that the 27% of included articles has a low risk of bias, 55% a medium risk of bias and 18% a high risk of bias. With the non-randomized control studies (8 of the 19 studies) the most frequently occurring source (100%) of bias appeared to be selection of participants. Additionally, approximately 60% of the articles either did not report on issues related to loss of follow-up or did not adequately describe and analyze this data. Additional information on the risk of bias assessment can be found in Supplementary file 2. No studies for excluded from the study on the basis of the bias assessment.

### 3.2 Intervention designs

14 studies recruited participants or were situated in clinical settings including pediatric clinics, planned parenthood centers and outpatient clinics (Bar-Shain et al., 2015, Cassidy et al., 2014, Dempsey, 2019, Dixon et al., 2019, Henrikson et al., 2018, Keeshin and Feinberg, 2017, Kempe et al., 2016, Kharbanda et al., 2011aa, Matheson et al., 2014, Patel et al., 2014, Rand et al., 2015, Richman et al., 2019, Richman et al., 2016, Szilagyi et al., 2013). Non-clinical based studies utilized multiple approaches, for example Tull et al. was conducted within schools and Aragones et al. recruited in the non-clinical environment of an embassy health window. Some studies focused on specific sub-populations such as Koreans (Lee et al., 2016), Latinos (Dempsey, 2019, Dixon et al., 2019), low income or socio-economic status adolescents (Bar-Shain et al., 2015, Richman et al., 2019, Szilagyi et al., 2013), people living with HIV (Keeshin and Feinberg, 2017)and college students (Richman et al., 2016).

The most frequent aim of the interventions included was based on the assumption that reminding parents, young adults or adolescents of their intervention schedule would increase intervention uptake. This is evidenced by the utilization of reminder systems by 16 of the included studies. Ten studies utilized text-message reminder systems. Nine of these studies assessed a reminder that the adolescent enrolled in the study was due for vaccination and asked the receiver to schedule an appointment with the health care provider (Bar-Shain et al., 2015, Henrikson et al., 2018, Keeshin and Feinberg, 2017, Kharbanda et al., 2011aa, Matheson et al., 2014, Morris et al., 2015, Patel et al., 2014, Rand et al., 2015, Rand et al., 2017). However, the tenth study Tull et al. (Tull et al., 2019), made a distinction assessed the differential outcomes of two different types of text-message reminders: motivational texts versus self-regulatory texts. (Aragones et al., 2015). The other studies utilized phone call based reminder (Cassidy et al., 2014).

Three studies did not utilize reminder but rather focused on improving knowledge and attitudes. Two of the 19 studies utilized mobile applications displayed on tablets in pediatric clinic waiting and examination rooms (Dempsey, 2019, Dixon et al., 2019). Lee et al. developed text messages which were geared at improving knowledge and attitudes. An overview of the intervention designs is found in Table 2.

**Table 2:** mhealth intervention design.

**Author**	**Intervention Description**
Aragones et al. (Aragones et al., 2015)	• Parental education sessions on HPV information were conducted by lay health workers • Parents received weekly text messages until the receipt of first dose was reported or six weeks elapsed
Bar-Shain et al. (Bar-Shain et al., 2015)	• Parents/guardians received messages via email, text message and phone call • The messages were delivered using stepwise cascade in which the order of messaging was email, then text messaged and a call • In cases with missing phone numbers, post cards were sent • The same message was sent to parents/guardians regardless of the messaging modality
Cassidy et al. (Cassidy et al., 2014)	• Parents first received a literature-based brochure • Afterwards, automated telephone calls were made to remind parents of their upcoming appointments • If patients failed to show-up at the second or third dose appointments, a non-automated phone call was made
Dempsey (Dempsey, 2019)	• The study utilized the “Combatting HPV Infections and Cancers (CHICOS)” which delivered tailored informational materials on an iPad • While at a doctor’s office, participants were invited to complete baseline surveys which included questions related to their attitudes and believes about HPV and HPV vaccinations. Additionally demographics and current vaccination dose completion was recorded • Based on the baseline information, CHICOS developed tailored information for parents, which they could review on an iPad • The information was presented in two languages (English and Spanish) and an assistant was available to offer help with either technology or content
Dixon et al. (Dixon et al., 2019)	• The study utilized two independent programs for two participant groups (those who had not received the first dose of the HPV vaccine and those who had received the first or second dose of the HPV vaccine and planned to complete the series) • Messaging for both programs were delivered in English and Spanish based on participants preferences
Henrikson et al. (Henrikson et al., 2018)	• In the first step of the study, parental concerns about HPV and barriers to vaccination were assessed • Then an intervention was then developed to address the identified needs and utilized a both mailed reminders and telephone/text reminders. • Participants first received a mailed outreach letter/brochure and consequently, automated reminders for the vaccination appointment were sent along with information related to financial and health center information • Eight weeks after the initial outreach letters, a reminder calls via interactive voice recognition were made, as well as reminder text messages with automated script were sent
Keeshin et al. (Keeshin and Feinberg, 2017)	• The intervention gave patients the option to receive months text message reminders or monthly emails. All patients opted for the text messages • Patients who had not completed of the three HPV vaccination doses, were sent monthly text messages until completion of series
Kempe et al. (Kempe et al., 2016)	• In this intervention, adolescents who were late in completing their vaccination series were followed up • Parents had the option to receive reminders via (text, email, automated telephone message) • Additionally, they could also opt-in to have reminders also sent to their child • The recall was sent on alternating weeks for up to three recalls per week for six weeks • The number of recalls sent depended on the number of methods selected for a total of six recalls were sent, one each week alternating between methods
Kharbanda et al. (Kharbanda et al., 2011aa, Kharbanda et al., 2011bb)	• When parents signed up to receive reminders and took the first dose a series, reminders were activated for the second and third doses • Messages were sent in English or Spanish based on participant preferences • For the three weeks prior to the vaccination due date, participants received three weekly reminders
Lee et al. (Lee et al., 2016)	• Community-based participatory research (CBPR) methods were utilized to develop text messages for Korean American women • The message content included information about HPV, HPV vaccination but also about cervical cancer and experiences of Korean American women • The developed messages were sent over a consecutive seven day period
Matheson et al. (Matheson et al., 2014)	• Participants in the intervention could receive three text message reminders for each of the three HPV vaccine does appointments. • The messages were sent 1)seven days prior to each HPV vaccination date, 2)the vaccine due date and 3) seven days after the due date • The content of the message was consistent in all three messages with the exception of including the relevant appointment date and an office number in the case of missed appointments
Morris et al (Morris et al., 2015)	• Participants could receive text, email or postal reminders • The intervention include three reminder phases, based on their vaccine completion status. • Participants received the first reminder two weeks after they were enrolled. This was followed with additional reminders every two weeks for three months after the first and second set of reminders
Patel et al. (Patel et al., 2014)	• ﻿Participant could receive automated reminders by text message, e-mail, phone call, private Facebook message, or standard mail. • In the six weeks after the first visit, participants received four reminders (or one if standard mail was the selected reminder method) • The reminders contained health center contact information for women to schedule their appointments
Rand et al. (Rand et al., 2015)	• Participants could receive telephone or text message reminders • Participants received three reminders to schedule their vaccination appointment (once a week) and again after six weekends if the vaccinations were not taken
Rand et al. (Rand et al., 2017)	• Participants could receive telephone or text message vaccine reminder • Participants could receive up to three reminders per dose (once a week) with up to six reminders if the vaccination was not taken
Richman et al. (Richman et al., 2016)	• Participants received reminder messages once a month for seven months • Four messages provided health education about HPV and the HPV vaccine, two of the messages were appointment reminders are one was an invitation for a follow-up study
Richman et al. (Richman et al., 2019)	• Participants received reminder messages once a month for seven months • Four messages provided health education about HPV and the HPV vaccine, two of the messages were appointment reminders are one was an invitation for a follow-up study
Szilagyi et al. (Szilagyi et al., 2013)	• Participants received either reminder letters or a phone call • The content and frequency of the messages was the same on both modalities and they advised parents to schedule their vaccination appointments as well as contact information for the health center • For the first vaccine done a reminder was sent over 10-week intervals and for vaccine dose 2 and 3 reminders were send over 5-week intervals with a maximum of 8 reminders per vaccine • Messaging was delivered in English and Spanish based on participants preferences • In addition to the HPV vaccination, reminders were also sent for the Tdap and MCV4 vaccine
Tull et al. (Tull et al., 2019)	• Parents/guardians received a motivational or a self-regulatory text message before the third dose vaccine was scheduled • Both the motivation and self-regulatory messages included the name of the provider, child, date of vaccine appointment at school • They differed in that the motivation message included some information on the problem of vaccine-preventable diseases in the community while the he self-regulatory messages prompted parents/guardians to make plans for the appointment day

There was no consistency in the timing and frequency of messages sent by studies. Aragones et.al sent text message reminders were sent once a week until reported uptake of the first vaccine or six weeks. Whereas other interventions like Szilagyi et.al and Patel et.al varied the reminder frequency based on the dose due. For example, prior to dose one, reminder calls were performed in 10-week intervals, whereas 5-week intervals were used prior to dose two and three (Szilagyi et al., 2013). Rand et al. (Rand et al., 2017) and Bar-Shain et al. (Bar-Shain et al., 2015) utilized both text-message and telephone reminders, which were repeatedly sent every two months if the vaccination did not take place. Cassidy et al. followed up with a standardized call when after a reminder call participants did not show up for their second or third vaccination appointment (Cassidy et al., 2014).

### 3.3 HPV knowledge and vaccination Intent

Only four of the included interventions specifically focused on HPV vaccination knowledge and intention (Dempsey et al., 2019, Lee et al., 2016, Richman et al., 2019, Richman et al., 2016). Lee et al. (Lee et al., 2016) found a statistically significant increase in knowledge and intent to vaccinate between intervention participants pre and post intervention. Richman et al. (Richman et al., 2016, Richman et al., 2019) found positive differences in knowledge in the intervention group between baseline and end line. However, these changes were only significant in Richman et al. (Richman et al., 2019). Dempsey et al. noted an increase in intent to vaccinate in the post-intervention study with no significant differences between control and intervention groups (Dempsey et al., 2019).

In regards to the mHealth intervention utilized, Richman (Richman et al., 2016, Richman et al., 2019) sent standard SMS messages, whereas Lee and Dempsey utilized tailored messages. Lee et al. (Lee et al., 2016) utilized culturally tailored SMS messages based on principles of community participatory research and the Fogg Behavior Model. The messages were tested with focus groups and delivered testimonies from peers about cervical cancer and the HPV vaccination, as well as providing information about locations for the vaccination and testing (Lee et al., 2016). Dempsey et al. (Dempsey et al., 2019) utilized tailored health messaging in clinic waiting rooms to reach Latino parents of adolescents or young adults aged 18–26 years (Dempsey et al., 2019).

### 3.4 HPV vaccination uptake

Of the ten studies which utilized text message reminders, nine reported an increased receipt of vaccination (Aragones et al., 2015, Bar-Shain et al., 2015, Henrikson et al., 2018, Keeshin and Feinberg, 2017, Kharbanda et al., 2011bb, Matheson et al., 2014, Rand et al., 2017, Rand et al., 2015, Tull et al., 2019). The one study which did not report any increases, proposed that their choice to target an unconventional population for HPV vaccinations, young adults aged 18 to 26 years old, could have been a limiting factor (Patel et al., 2014). Five of these studies reported high series completion rates (Aragones et al., 2015, Bar-Shain et al., 2015, Henrikson et al., 2018, Keeshin and Feinberg, 2017, Matheson et al., 2014, Rand et al., 2017). Aragones et al. reported that those in the text message group were 15.5 times more likely to complete the vaccination than those in the education-only group (p < 0.001) (Aragones et al., 2015). Bar-Shain et al (Bar-Shain et al., 2015) noted that parents who received one message (35.6%) were more likely to get vaccinated than those that received two (19.4%) or three messages (24.1%; p < 0.0001) (Bar-Shain et al., 2015). Tull et al. (2018) found that both forms of text messages, motivational and self-regulatory, resulted in an increase in HPV vaccine receipt with a slightly high point increase in the motivational group (3.29% vs 2.64%).

Six studies utilized telephone reminders (Bar-Shain et al., 2015, Cassidy et al., 2014, Henrikson et al., 2018, Patel et al., 2014, Rand et al., 2017, Szilagyi et al., 2013), of which only one assessed the effect of telephone reminders alone (Cassidy et al., 2014). This study found that 62.5% of participants who received reminder phone calls completed the vaccination series, compared to 6.9% in the control group. The study noted that even though vaccination rates are significantly higher, success rates cannot be attributed to the telephone reminders exclusively as parents intending to vaccinate their daughters might have been more likely to sign up for the telephone reminder systems (Cassidy et al., 2014). Overall, when assessing telephone reminders in comparison to other reminder systems, vaccination and completion rates did not differ. Szilagyi et al. (Szilagyi et al., 2013) observed increased vaccination rates of 53% in the telephone reminder group, compared to a group that received mailed reminders (56%). In this study, the lack of accurate telephone numbers limited the potential reach of participants. In comparison to text-message reminders, one study reported that significantly less participants with telephone reminders completed the vaccination series (Rand et al., 2017). The remaining three studies including telephone reminders did not identify any differences in receipt of vaccination (Bar-Shain et al., 2015, Henrikson et al., 2018; Patel et al., 2014).

Regarding web-based interventions, Dixon et al. (Dixon et al., 2019), observed that providing mobile tablets to parents waiting in the examination room of pediatric clinics, resulted in an increase in vaccination rates of the intervention group (78%) compared to the control group (52.8%). The utilized application was interactive and provided one of two specific programs based on the adolescent’s HPV vaccination status (Dixon et al., 2019). Dempsey et al. (Dempsey et al., 2019) found no statistically significant differences in the intention to receipt of the vaccination among the groups that received the tailored information compared to untailored or standard care. (Dempsey, 2019).

## 4 Discussion

HPV vaccination rates remain suboptimal across the globe, including in North American and European countries where vaccine availability is not a significant barrier (Bruni et al., 2016). As countries attempt to address the low uptake of vaccinations, including HPV, there is a greater interest in digital technologies, including mHealth, as a possible solution (Dumit et al., 2018, Francis et al., 2017, Oliver-Williams et al., 2017a). This review sought to synthesize existing evidence on mHealth interventions geared at improving HPV related knowledge, vaccination intent and vaccination uptake. The findings suggest that mHealth interventions can be successfully utilized to improve short-term HPV knowledge, intent to vaccinate and vaccination uptake but there are limitations.

The 19 studies included provided a range of mHealth interventions, including standard text messages, interactive voice messages to tailored programs delivered on a tablet. Despite this variation in intervention designs, all but five studies (Dempsey et al., 2019, Patel et al., 2014, Richman et al., 2016, Richman et al., 2019; Szilagyi et al., 2013) reported increases in knowledge, intent to vaccinate or vaccination uptake. However, this cannot be linked to any specific format of mHealth intervention or any theoretical underpinnings. Overall, the majority of the included studies did not elucidate on their theoretical understanding of health education, health communication, mHealth intervention development or adoption.

In this review, only three interventions attempted to utilize culturally developed or tailored messages deliver health education information (Dempsey et al., 2019, Dixon et al., 2019, Lee et al., 2016). In communities and countries where vaccination rates are related to issues around scheduling and remembering to vaccinate, simple text messages or appointment phone call reminders might be sufficient to see an increase in uptake. However, in contexts where there is active vaccine denial due to lack of knowledge, prevailing attitudes or misinformation, more targeted or tailored health interventions with clear theoretical underpinnings are needed (Barnard et al., 2017, Beavis et al., 2017, Gordon et al., 2011, Holman et al., 2014, Kester et al., 2013, Loke et al., 2017). Recent reviews have explored the role of existing psychological and health behavior theories on the development of effective mHealth interventions (Morrison, 2015, Riley et al., 2011). In addition to the need to fully engage with theory, there is additional evidence which suggests that targeting or tailoring health messages to populations or individuals who are less well-informed or hold negative attitudes towards a health intervention could lead to improved health outcomes (Kreuter et al., 2012, Wanyonyi et al., 2011).

Most of the studies targeted parents and the two studies which targeted young adults reported contrasting results. A recent review by Barnard et al. (Barnard et al., 2019) which focused on interventions for college students found few improvements in vaccination uptake. While the vaccination is recommended to be given before the onset at sexual activity and is generally given between 9 and 13, there is evidence that taking the vaccine before the age of 26 still confers protective effects (Ault, 2007). In Barnard’s review of nine articles, only one of the interventions utilized an mHealth intervention (Richman et al., 2016). Considering the growing technological awareness and engagement among adolescents and young adults, not targeting adolescents and young adults in mHealth interventions geared at improving HPV knowledge, intent and vaccination might be a missed opportunity. This, of course, must include the consideration that while in some contexts adolescents may be independent decision-makers about vaccinations, this is not the case in most countries. However, there is some discussion in countries like the US about the need to give adolescents decision-making power in relation to vaccinations (Silverman et al., 2019).

Another interesting point for consideration raised by this review is the possibility for interventions with mixed information delivery modes. In this review, a number of interventions combined two mHealth interventions, for example, texting and phone calls. However, Cassidy et al. (2014) actually combined evidence-based health education sessions with a text/phone reminder and noted statistically significant improvements in vaccination uptake. Also, Lee et al. (Lee et al., 2016) utilized principles of community-based participatory research to achieve successful outcomes, albeit in a small population. These two studies exemplify the earlier point on the need for theoretical bases in mHealth research and also on the potential for innovation in the design, implementation, and adoption of mHealth. Adoption of mHealth is known to be influenced by methods used in the development process of the intervention, including creating ownership among the target population. One way to do this is through the utilization of co-creation methods and usability assessments in the development and pilot testing phases of mHealth. This approach of combining intervention modalities and adopting theoretically driven intervention design could also be of significant importance in addressing issues related to the *digital divide*. This is pertinent to ensuring that a reliance on technological interventions does not worsen disparities in health access. It has been established that a digital divide exists along lines of age, ethnicities and sociodemographic characteristics, thus paying attention to known sub-population needs and trends could ensure that the increased attention to digital interventions contributes to the improvement of health outcomes for all.

Finally, the main limitation of this review, is the poor geographical representation among included studies. All but one intervention was conducted in the United States. This is a very important point in the interpretation of the review results. While the results are promising, the design, funding and availability of HPV vaccination vary greatly between countries, even high-income countries. In some countries, HPV vaccination has been included in the national vaccination scheme and thus the practicalities of receiving the vaccination differ from countries where the vaccination is recommended. Additionally, countries handle the vaccination of males differently. Some countries such as Australia, Canada, the United States and Germany, have adapted HPV vaccination programs to include males (Al Romaih et al., 2011, Brill, 2013, Quinn and Goldman, 2015). However, this is not the case in every country. Additionally, factors including norms and values, culture, general attitudes towards vaccination and ethnic diversity of targeted populations, also have the potential to greatly influence the reception, implementation, and outcome of an mHealth intervention. Additionally, publication bias could be an additional limitation in this review, given that the majority of studies reported positive if not significant findings. However, it is good to note that there were also a couple of included articles which also found no improvements in the intervention group. Finally, the risk of bias assessment indicated that while some articles presented a low risk of bias, the majority of the studies could not be conclusively considered low risk.

Therefore, while the results of this review are overwhelmingly positive, they should be interpreted with caution in varying contexts. It is also of note that the majority of the included studies targeted minority groups such as Latinos or socio-economically disadvantaged groups. This could indicate that the study findings could potentially be replicated also in ethnically and socioeconomically diverse groups within and outside the United States.

## 5 Conclusion

Within the context of the United States, mHealth interventions have shown great potential for improving rates of vaccination. However, a better understanding of the theories that can contribute to an effective mHealth intervention is needed, alongside testing of mHealth interventions in different contexts and amongst diverse population groups.

## Author contributions

The review was designed and performed by OI and PS with support from JK and MJ. JK developed the search strings utilized in all databases. OI played a lead role in all stages of the review and in drafting the manuscript, seconded by PS, with JK and MJ contributing to its revision. All authors have read and approved the final manuscript.

## Declaration of Competing Interest

The authors declare that they have no known competing financial interests or personal relationships that could have appeared to influence the work reported in this paper.
