# Supplementary material for: Can mHealth interventions contribute to increased HPV vaccination uptake? A systematic review
Source: Prev Med Rep. 2020 Dec 28;21:101289. doi: 10.1016/j.pmedr.2020.101289 (PMC7777527; doi:10.1016/j.pmedr.2020.101289)
Supplement: Supplementary data 1 [file mmc1.docx]

**Ilozumba Schmidt Supplementary File 1 20200626**

**Supplementary File 1: Search strategies per database**

**Search strategy for PubMed (5 November 2019)**

| **Search** | **Query** | **Items found** |
| --- | --- | --- |
| #4 | #1 AND #2 AND #3 | 276 |
| #3 | "Reminder Systems"[Mesh] OR "Cell Phone"[Mesh] OR "Telemedicine"[Mesh] OR "Telenursing"[Mesh] OR "Computers, handheld"[Mesh] OR "Mobile Applications"[Mesh] OR "Internet"[Mesh] OR mhealth*[tiab] OR m health*[tiab] OR telehealth*[tiab] OR tele health[tiab] OR ehealth*[tiab] OR e-health*[tiab] OR app[tiab] OR apps[tiab] OR smartphone*[tiab] OR smart phone*[tiab] OR phone application*[tiab] OR telephone application*[tiab] OR health application*[tiab] OR ipad[tiab] OR ipads[tiab] OR sms[tiab] OR mms[tiab] OR text messag*[tiab] OR texting[tiab] OR ussd[tiab] OR mhapps[tiab] OR iphone*[tiab] OR android[tiab] OR whatsapp*[tiab] OR social media*[tiab] OR social network*[tiab] OR facebook*[tiab] OR instagram*[tiab] OR reddit[tiab] OR twitter*[tiab] OR youtube[tiab] OR digital*[tiab] OR mobile[tiab] OR cell phone*[tiab] OR clue[tiab] OR “tik tok”[tiab] OR tiktok[tiab] OR snapchat*[tiab] OR “snap chat”[tiab] OR spotify[tiab] OR imusic[tiab] OR viber[tiab] OR weibo[tiab] OR linkedin[tiab] OR “linked in”[tiab] | 397,135 |
| #2 | "Vaccination"[Mesh] OR vaccin*[tiab] OR immunizati*[tiab] OR immunisati*[tiab] OR inoculati*[tiab] | 426,102 |
| #1 | "Papillomaviridae"[Mesh] OR human papilloma vir*[tiab] OR hpv[tiab] OR hpvs[tiab] OR papillomavir*[tiab] OR alphapapillomavir*[tiab] OR betapapillomavir*[tiab] OR gammapapillomavir*[tiab] OR mupapillomavir*[tiab] | 55,436 |

**Search strategy for Clarivate Analytics/Web of Science** **Social Science Citation Index (SSCI) (20 November 2019)**

| **Set** | **Query** | **Results** |
| --- | --- | --- |
| **#4** | #3 AND #2 AND #1 | **142** |
| **#3** | TOPIC: (“mhealth*” OR “m health*” OR “telehealth*” OR “tele health” OR “ehealth*” OR “e-health*” OR “app” OR “apps” OR “smartphone*” OR “smart phone*” OR “phone application*” OR “telephone application*” OR “health application*” OR “ipad” OR “ipads” OR “sms” OR “mms” OR “text messag*” OR “texting” OR “ussd” OR “mhapps” OR “iphone*” OR “android” OR “whatsapp*” OR “social media*” OR “social network*” OR “facebook*” OR “instagram*” OR “reddit” OR “twitter*” OR “youtube” OR “digital*” OR “mobile” OR “cell phone*” OR “clue” OR “tik tok” OR “tiktok” OR “snapchat*” OR “snap chat” OR “spotify” OR “imusic” OR “viber” OR “weibo” OR “linkedin” OR “linked in”) | **137,435** |
| **#2** | TOPIC: (“vaccin*” OR “immunizati*” OR “immunisati*” OR “inoculati*”) | **23,543** |
| **#1** | TOPIC: (“human papilloma vir*” OR “hpv” OR “hpvs” OR “papillomavir*” OR “alphapapillomavir*” OR “betapapillomavir*” OR “gammapapillomavir*” OR “mupapillomavir*”) | **5,456** |

**Search strategy for Ebsco/CINAHL (20 November 2019)**

| **#** | **Query** | **Results** |
| --- | --- | --- |
| **S7** | S5 AND S6 | **280** |
| **S6** | (MH "Reminder Systems") OR (MH "Cellular Phone") OR (MH "Text Messaging") OR (MH "Smartphone") OR (MH "Voice Mail") OR (MH "Telemedicine") OR (MH "Telenursing") OR (MH "Computers, Hand-Held") OR (MH "Mobile Applications") OR (MH "Internet") OR (MH "Email") OR (MH "Social Media+") OR (MH "Instant Messaging") OR TI(mhealth* OR “m health*” OR telehealth* OR “tele health” OR ehealth* OR “e-health*” OR app OR apps OR smartphone* OR “smart phone*” OR “phone application*” OR “telephone application*” OR “health application*” OR ipad OR ipads OR sms OR mms OR “text messag*” OR texting OR ussd OR mhapps OR iphone* OR android OR whatsapp* OR “social media*” OR “social network*” OR facebook* OR instagram* OR reddit OR twitter* OR youtube OR digital* OR mobile OR “cell phone*” OR clue OR “tik tok” OR tiktok OR snapchat* OR “snap chat” OR spotify OR imusic OR viber OR weibo OR linkedin OR “linked in”) OR AB(mhealth* OR “m health*” OR telehealth* OR “tele health” OR ehealth* OR “e-health*” OR app OR apps OR smartphone* OR “smart phone*” OR “phone application*” OR “telephone application*” OR “health application*” OR ipad OR ipads OR sms OR mms OR “text messag*” OR texting OR ussd OR mhapps OR iphone* OR android OR whatsapp* OR “social media*” OR “social network*” OR facebook* OR instagram* OR reddit OR twitter* OR youtube OR digital* OR mobile OR “cell phone*” OR clue OR “tik tok” OR tiktok OR snapchat* OR “snap chat” OR spotify OR imusic OR viber OR weibo OR linkedin OR “linked in”) OR KW(mhealth* OR “m health*” OR telehealth* OR “tele health” OR ehealth* OR “e-health*” OR app OR apps OR smartphone* OR “smart phone*” OR “phone application*” OR “telephone application*” OR “health application*” OR ipad OR ipads OR sms OR mms OR “text messag*” OR texting OR ussd OR mhapps OR iphone* OR android OR whatsapp* OR “social media*” OR “social network*” OR facebook* OR instagram* OR reddit OR twitter* OR youtube OR digital* OR mobile OR “cell phone*” OR clue OR “tik tok” OR tiktok OR snapchat* OR “snap chat” OR spotify OR imusic OR viber OR weibo OR linkedin OR “linked in”) | **215,030** |
| **S5** | S3 OR S4 | **6,282** |
| **S4** | S1 AND S2 | **5,244** |
| **S3** | MH "Papillomavirus Vaccine" | **4,452** |
| **S2** | MH "Immunization+" OR TI(vaccin* OR immunizati* OR immunisati* OR inoculati*) OR AB(vaccin* OR immunizati* OR immunisati* OR inoculati*) OR KW(vaccin* OR immunizati* OR immunisati* OR inoculati*) | **57,654** |
| **S1** | MH "Papillomaviruses" OR MH "Papillomavirus Infections" OR TI(“human papilloma vir*” OR hpv OR hpvs OR papillomavir* OR alphapapillomavir* OR betapapillomavir* OR gammapapillomavir* OR mupapillomavir*) OR AB(“human papilloma vir*” OR hpv OR hpvs OR papillomavir* OR alphapapillomavir* OR betapapillomavir* OR gammapapillomavir* OR mupapillomavir*) OR KW(“human papilloma vir*” OR hpv OR hpvs OR papillomavir* OR alphapapillomavir* OR betapapillomavir* OR gammapapillomavir* OR mupapillomavir*) | **14,566** |

**Search strategy for Ebsco/PsycInfo (20 November 2019) (107)**

| **#** | **Query** | **Results** |
| --- | --- | --- |
| **S4** | S1 AND S2 AND S3 | **107** |
| **S3** | DE "Online Social Networks" OR DE "Social Media" OR DE "Mobile Phones" OR DE "Smartphones" OR DE "Mobile Applications" OR DE "Wearable Devices" OR DE "Mobile Devices" OR DE "Mobile Health" OR DE "Text Messaging" OR DE "Telemedicine" OR DE "Internet" OR DE "Tablet Computers" OR DE "Computer Mediated Communication" OR TI(mhealth* OR “m health*” OR telehealth* OR “tele health” OR ehealth* OR “e-health*” OR app OR apps OR smartphone* OR “smart phone*” OR “phone application*” OR “telephone application*” OR “health application*” OR ipad OR ipads OR sms OR mms OR “text messag*” OR texting OR ussd OR mhapps OR iphone* OR android OR whatsapp* OR “social media*” OR “social network*” OR facebook* OR instagram* OR reddit OR twitter* OR youtube OR digital* OR mobile OR “cell phone*” OR clue OR “tik tok” OR tiktok OR snapchat* OR “snap chat” OR spotify OR imusic OR viber OR weibo OR linkedin OR “linked in”) OR AB(mhealth* OR “m health*” OR telehealth* OR “tele health” OR ehealth* OR “e-health*” OR app OR apps OR smartphone* OR “smart phone*” OR “phone application*” OR “telephone application*” OR “health application*” OR ipad OR ipads OR sms OR mms OR “text messag*” OR texting OR ussd OR mhapps OR iphone* OR android OR whatsapp* OR “social media*” OR “social network*” OR facebook* OR instagram* OR reddit OR twitter* OR youtube OR digital* OR mobile OR “cell phone*” OR clue OR “tik tok” OR tiktok OR snapchat* OR “snap chat” OR spotify OR imusic OR viber OR weibo OR linkedin OR “linked in”) OR KW(mhealth* OR “m health*” OR telehealth* OR “tele health” OR ehealth* OR “e-health*” OR app OR apps OR smartphone* OR “smart phone*” OR “phone application*” OR “telephone application*” OR “health application*” OR ipad OR ipads OR sms OR mms OR “text messag*” OR texting OR ussd OR mhapps OR iphone* OR android OR whatsapp* OR “social media*” OR “social network*” OR facebook* OR instagram* OR reddit OR twitter* OR youtube OR digital* OR mobile OR “cell phone*” OR clue OR “tik tok” OR tiktok OR snapchat* OR “snap chat” OR spotify OR imusic OR viber OR weibo OR linkedin OR “linked in”) | **200,671** |
| **S2** | DE "Immunization" OR TI(vaccin* OR immunizati* OR immunisati* OR inoculati*) OR AB(vaccin* OR immunizati* OR immunisati* OR inoculati*) OR KW(vaccin* OR immunizati* OR immunisati* OR inoculati*) | **9,826** |
| **S1** | DE "Human Papillomavirus" OR TI(“human papilloma vir*” OR hpv OR hpvs OR papillomavir* OR alphapapillomavir* OR betapapillomavir* OR gammapapillomavir* OR mupapillomavir*) OR AB(“human papilloma vir*” OR hpv OR hpvs OR papillomavir* OR alphapapillomavir* OR betapapillomavir* OR gammapapillomavir* OR mupapillomavir*) OR KW(“human papilloma vir*” OR hpv OR hpvs OR papillomavir* OR alphapapillomavir* OR betapapillomavir* OR gammapapillomavir* OR mupapillomavir*) | **1,881** |
