# Supplementary material for: Can mHealth interventions contribute to increased HPV vaccination uptake? A systematic review
Source: Prev Med Rep. 2020 Dec 28;21:101289. doi: 10.1016/j.pmedr.2020.101289 (PMC7777527; doi:10.1016/j.pmedr.2020.101289)
Supplement: Supplementary data 2 [file mmc2.docx]

Table 1: Cochrane Risk of Bias Assessment

| Author and Year | Randomization process | Deviations from intended interventions | Missing outcome data | Measurement of the outcome | Selection of the reported result | Overall |  |  |
| --- | --- | --- | --- | --- | --- | --- | --- | --- |
| Henrikson et al. 2018 |  |  |  |  |  |  |  | Low risk |
| Dempsey et al. 2018 |  |  |  |  |  |  |  | Some concerns |
| Dixon et al. 2019 |  |  |  |  |  |  |  | High risk |
| Kempe et al.2016 |  |  |  |  |  |  |  |  |
| Patel et al.2014 |  |  |  |  |  |  |  |  |
| Rand et al. 2015 |  |  |  |  |  |  |  |  |
| Rand et al.2017 |  |  |  |  |  |  |  |  |
| Richman et al.2019 |  |  |  |  |  |  |  |  |
| Richman et al. 2016 |  |  |  |  |  |  |  |  |
| Szilagyi et al. 2013 |  |  |  |  |  |  |  |  |
| Tull et al. 2019 |  |  |  |  |  |  |  |  |

Table 2: Summary of Risk for RCT Studies

|  | **Randomization process** | **Deviations from intended interventions** | **Mising outcome data** | **Measurement of the outcome** | **Selection of the reported result** | **Overall Bias** |
| --- | --- | --- | --- | --- | --- | --- |
| Assignment to intervention (the 'intention-to-treat' effect) | | | |  |  |  |
| Total number of study = 11 | |  |  |  |  |  |
| Low risk | 72.7 | 63.6 | 63.6 | 45.5 | 45.5 | 27.3 |
| Some concerns | 18.2 | 27.3 | 18.2 | 54.5 | 36.4 | 54.5 |
| High risk | 9.1 | 9.1 | 18.2 | 0 | 18.2 | 18.2 |

Table 3: Joanna Briggs Appraisal of Analytical Cross-sectional Studies

| ***Appraisal Question*** | Aragones et al. (2015) | Bar-Shain et al. (2015) | Kharbanda et al. (2011) | Lee et al. (2016) | Matheson et al. (2014) | Cassidy et al. (2014) | Morris et al. (2015) | Keeshin et al. (2017) |
| --- | --- | --- | --- | --- | --- | --- | --- | --- |
| Is it clear in the study what is the ‘cause’ and what is the ‘effect’ | Yes | Yes | Yes | Yes | Yes | Yes | Yes | Yes |
| Were the participants included in any comparisons similar? | Yes | n/a | No | Yes | Unclear | n/a | No | No |
| Were the participants included in any comparisons receiving similar treatment/care, other than the exposure or intervention of interest? | Yes | n/a | Yes | No | Yes | n/a | Yes | Yes |
| Was there a control group? | No | No | Yes | No | Yes | Yes | Yes | Yes |
| Were there multiple measurements of the outcome both pre and post the intervention/exposure? | Yes | Yes | Yes | Yes | No | Yes | Yes | Yes |
| Was follow up complete and if not, were differences between groups in terms of their follow up adequately described and analyzed? | Yes | No | Yes | Yes | No | n/a | Unclear | No |
| Were the outcomes of participants included in any comparisons measured in the same way? | Yes | n/a | Yes | Yes | Yes | Yes | Yes | Yes |
| Were outcomes measured in a reliable way? | Yes | Yes | Yes | Yes | Yes | Yes | Yes | Yes |
| Was appropriate statistical analysis used | Yes | Yes | Yes | Yes | Yes | Yes | Yes | Yes |
